# Hepatitis C risk perceptions and attitudes towards reinfection among HIV‐diagnosed gay and bisexual men in Melbourne, Australia

**DOI:** 10.1002/jia2.25288

**Published:** 2019-05-21

**Authors:** Sophia E Schroeder, Peter Higgs, Rebecca Winter, Graham Brown, Alisa Pedrana, Margaret Hellard, Joseph Doyle, Mark Stoové

**Affiliations:** ^1^ Disease Elimination Program Burnet Institute Melbourne Australia; ^2^ Department of Public Health La Trobe University Melbourne Australia; ^3^ Department of Gastroenterology and Hepatology St Vincent's Hospital Melbourne Australia; ^4^ School of Public Health and Preventive Medicine Monash University Melbourne Australia; ^5^ Department of Infectious Diseases The Alfred Hospital Melbourne Australia

**Keywords:** hepatitis C, HIV, gay and bisexual men, direct‐acting antivirals, reinfection, risk behaviours, attitudes

## Abstract

**Introduction:**

Gay and bisexual men (GBM) are at increased risk of hepatitis C/HIV co‐infection. In Australia, the availability of subsidized direct‐acting antiviral treatment for hepatitis C has rendered eliminating co‐infection possible. High reinfection rates in subgroups with continued exposure may compromise elimination efforts. To inform the development of hepatitis C risk reduction support in GBM, we explored reinfection risk perceptions and attitudes among GBM living with HIV recently cured from hepatitis C.

**Methods:**

Between April and August 2017, 15 GBM living with diagnosed HIV were recruited from high caseload HIV primary care services in Melbourne following successful hepatitis C treatment. In‐depth interviews were conducted exploring understandings of hepatitis C risks, experiences of co‐infection and attitudes towards reinfection. Constructivist grounded theory guided data aggregation.

**Results:**

Participants’ understandings of their hepatitis C infection and reinfection trajectories were captured in three categories. *Hepatitis C and HIV disease dichotomies:* Hepatitis C diagnosis was a shock to most participants and contrasted with feelings of inevitability associated with HIV seroconversion. While HIV was normalized, hepatitis C was experienced as highly stigmatizing. Despite injecting drug use, interviewees did not identify with populations typically at risk of hepatitis C. *Risk environments and avoiding reinfection*: Interviewees identified their social and sexual networks as risk‐perpetuating environments where drug use was ubiquitous and higher risk sex was common. Avoiding these risk environments to avoid reinfection resulted in community disengagement, leaving many feeling socially isolated. *Hepatitis C care as a catalyst for change*: Engagement in hepatitis C care contributed to a better understanding of hepatitis C risks. Interviewees were committed to applying their improved competencies around transmission risk reduction to avoid reinfection. Interviewees also considered hepatitis C care as a catalyst to reduce their drug use.

**Conclusions:**

Hepatitis C/HIV co‐infection among GBM cannot be understood in isolation from co‐occurring drug use and sex, nor as separate from their HIV infection. Hepatitis C prevention must address subcultural heterogeneity and the intersectionality between multiple stigmatized social identities. Hepatitis C care presents an opportunity to provide support beyond cure. Peer support networks could mitigate social capital loss following a commitment to behaviour change and reduce hepatitis C reinfection risks.

## 1 Introduction

Hepatitis C virus (HCV) infection is a significant public health threat; in 2015, an estimated 71 million people worldwide had chronic HCV infection 1. Due to shared modes of transmission, HCV is a common co‐infection in people living with HIV (PLHIV), and roughly 2.3 million individuals worldwide are co‐infected, equating to a global HCV prevalence of 6.2% in the population living with HIV 2. Risk of HCV infection is estimated to be 1.6 times higher among PLHIV than in the HIV‐negative population, and this risk is six times higher in PLHIV with a history of injecting drug use and 7.5 times higher in HIV‐diagnosed gay and bisexual men (GBM) 2, 3. Reinfection post‐successful HCV treatment is also common in this group, with reinfection rates estimated to be 20 times that of primary infection rates 4.

Hepatitis C virus infection is particularly prevalent among HIV‐positive GBM practising multiple risk behaviours, such as methamphetamine use and participation in group sex 5, 6, 7, 8. Over the past decade, rising incidences of HCV have been observed among GBM living with diagnosed HIV in Asia 9, Europe 7, 8, 10, 11, North America 5, 12 and Australia 13, many of whom do not report a history of injecting drug use. While it is likely that HCV is introduced into sexual networks through injecting drug use 14, selection of partners on the basis of HIV status (serosorting) and certain sexual practices, including sexualized drug use (also known as “chemsex”), can then concentrate ongoing sexual and injecting drug‐related HCV transmissions among GBM living with HIV 15. The stigma attached to HCV – related to associations with injecting drug use, negative stereotypes about “drug users,” the chronicity of HCV infection, and its infectiousness 16, 17 – can exacerbate these behavioural risks by limiting dialogue and contributing to poor knowledge of HCV in gay communities that drives low risk perception, suboptimal testing and undiagnosed HCV infection 15, 18, 19, 20, 21, 22.

All‐oral direct‐acting antiviral agents (DAAs), which entered the market in 2013 to 2014 23, have revolutionized HCV management. DAAs are equally effective in HCV/HIV coinfection and HCV monoinfection, with cure rates of over 90% enabling significant reduction in the burden of HCV‐related morbidity and mortality among PLHIV 24, 25. In Australia, publicly subsidized DAA treatment was made available from 1 March 2016 to anyone living with HCV, regardless of disease progression and ongoing risk behaviours 26, 27, 28, making possible the elimination of HCV as a public health threat by 2030 26, 29, 30, 31. A concentrated epidemic of HCV infection among GBM living with diagnosed HIV means that scale‐up of DAA treatment in this population could lead to substantial reductions in HCV transmission 32, facilitating elimination 33. However, evidence of high rates of HCV reinfection among “sexually adventurous” GBM with recurring exposure in the interferon treatment era 4, 10, 34, 35 suggests that applying a treatment‐as‐prevention approach on its own may not be sufficient to achieve elimination of HCV/HIV co‐infection among GBM. This underscores the importance of implementing strategies for treatment support for risk reduction and routine HCV testing (and retreatment) in specific subgroups at risk of HCV reinfection 10, 35, 36.

HCV risk reduction and health promotion strategies used to prevent HCV infection and reinfection among people who inject drugs (PWID) 37, 38 do not reflect the needs or breadth of social and behavioural risk experienced by GBM. To inform the development of tailored HCV treatment support strategies to reduce HCV reinfection risk among GBM, we explored the experiences of GBM living with diagnosed HIV recently treated and cured of their HCV, including their HCV risk perceptions and attitudes towards reinfection.

## 2 Methods

### 2.1 Study design

We conducted in‐depth interviews with GBM recently cured of HCV and adopted constructivist grounded theory methods to guide iterative data collection and analysis. This methodology assumes the social construction of reality and aims to maintain participants’ voice in producing a conceptual interpretation of the social processes under study 39, 40, 41. The study was embedded in an open‐label non‐randomized clinical trial of HCV treatment for PLHIV 42. Based in Melbourne, Australia, the *Eliminating hepatitis C transmission by enhancing care and treatment among HIV co‐infected individuals* study (co‐EC) aims to demonstrate the HCV prevention impact of scaling up HCV treatment in individuals co‐infected with HIV and assess the feasibility of micro‐elimination in the population co‐infected with HCV/HIV through a treatment‐as‐prevention approach 43. Co‐EC aimed to treat 75% of all GBM living with HCV/HIV co‐infection in the state of Victoria (approximately 375 individuals) over 18 months from its commencement in April 2016 44.

### 2.2 Sampling and recruitment

We adopted a consecutive sampling strategy of all eligible participants who attended post‐HCV treatment visits (SVR12 or SVR24) between April and August 2017 at three high caseload HIV clinics in Melbourne. Eligibility criteria included male gender, self‐identifying as gay or bisexual, living with diagnosed HIV and having recently completed DAA treatment and achieved HCV cure (sustained virological response after 12 or 24 weeks). Interested participants were telephoned by the first author to arrange face‐to‐face interviews in mutually convenient locations. Of the 22 eligible men who presented to the clinic over the recruitment period, 17 agreed to be interviewed but two were later unavailable, resulting in a total of 15 interviews.

### 2.3 Data collection

We developed a semi‐structured interview schedule by reviewing literature, drawing on the expertise of HCV and HIV researchers and consulting GBM researchers. The schedule was pilot tested with two GBM community members living with HIV. Questions focused on HCV awareness and experiences of HCV care, living with HCV/HIV co‐infection and participants’ understanding of and attitudes towards HCV reinfection risk. Prior to being interviewed, a written informed consent was obtained from all participants. Interviews took a median of 63 minutes (range 30 to 80 minutes), were digitally recorded and complemented by interviewer field notes. In accordance with standard research practice, participants were reimbursed AUD40 for their time and expertise.

### 2.4 Data management and analysis

The first author transcribed each interview verbatim and preliminary inductive analyses informed subsequent interviews. Identifying information was removed and pseudonyms assigned. Transcripts were uploaded into a qualitative software program (NVivo 11) for analysis. Following constructivist grounded theory methods 39, during initial coding the first author analysed all transcripts line by line and developed descriptive codes that defined the data. Next, codes were sorted and synthesized and connections between them were identified to elucidate higher level categories. Constant comparisons between the interview transcripts, the codes and the categories ensured that the generated categories were grounded in the data 45. Fortnightly, debriefings with co‐investigators supported researcher reflexivity, refinement of research design and further interpretation of results.

The study was approved by the Alfred Hospital Ethics Committee.

## 3 Results

### 3.1 Participant characteristics

Fifteen participants, aged 26 to 60 years (median 46 years), were interviewed. With the exception of one participant who identified as bisexual, all participants identified as gay. Participants had been living with diagnosed HIV for a median time of 15 years (range 2 to 29 years). Diagnostic HCV testing was prompted primarily by irregularities in liver function which was assessed as part of HIV management. The median time since HCV diagnosis was seven years (range one to twenty‐three years). Twelve participants had used illicit drugs in their lifetime and 10 reported ever injecting drugs; these characteristics broadly reflect the characteristics of the co‐EC cohort 46.

### 3.2 HCV and HIV disease dichotomies

Interviewees commonly used HIV as a point of comparison when describing their experiences with HCV. Despite lingering stigma, participants perceived HIV as normalized within the gay community and a manageable illness. Several interviewees discussed that an HIV diagnosis “means nothing these days” (Sean, 39 years) and those more recently diagnosed had accepted HIV infection as an inevitable consequence of their lifestyles:

*I'd already come to terms with [HIV] before I was diagnosed. I was like cool, the way that I'm living my life at the moment it's going to happen… I've always known that it wasn't so much a death sentence as a treatable disease* (Liam, 26).

It was suggested that prevention messages among gay men are generally focused on HIV at the expense of other threats to wellbeing, including HCV. As Michael (48) pointed out: *In the gay community there's a lot of hype about HIV and protecting yourself but there wasn't really much about hep C so I was a bit ignorant of it*.

Since being diagnosed with HIV most had limited their sexual networks to other PLHIV and HIV disclosure formed a natural – albeit uncomfortable – part of sexual negotiations. In contrast, HCV was experienced as *a taboo thing* (Raymond, 60) resulting from HCV‐related stigma and a lack of information about sexual transmission risks:

*I knew that [hepatitis] C existed and was one that injecting drug users tended to have, but I thought that was more associated with heroin users who were desperate and sharing needles* (Angelo, 48).

The HCV diagnosis came as a shock to almost everyone and evoked feelings of shame, which interviewees associated with contracting a virus they perceived as more stigmatizing than HIV. The contrasting responses between HCV diagnosis and HIV infection illustrated community stigma around injecting drug use and PWID which extended to internalized stigma and label avoidance:

*It was bad enough when I found out I was HIV positive but when I found out that I had hep C… I felt disgusted, like I was a disease, and judged, and felt dirty and gross… and where did I catch it from?* (Jeremy, 31)

Despite injecting drug use being prevalent – six participants reported currently injecting – a preference for using stimulants in sexual and/or party settings was used by participants to distinguish themselves from more stereotypical injecting drug users outside of the gay community whom they perceived as being at risk of HCV infection. As a result of risk perceptions centred largely on the sharing of needles by people who inject drugs, HCV did not feature in the interviewees’ general risk analyses:

*I perceived it as not really a risk worth being concerned about or mentioning unless you're doing the activities that you believe are at risk* [for HCV transmission], *which is sharing drugs or needles* (Joshua, 38).

To make sense of their diagnosis, some blamed a lack of awareness of risk reduction strategies related to sharing other drug paraphernalia, but most pointed to sexual HCV exposure as their likely mode of transmission:

*I was very confused* [when diagnosed with HCV]. *The closest running theory I've got is that it was somehow transmitted sexually. Because I didn't start injecting until I was already diagnosed* (Liam, 26).

### 3.3 Risk environments and avoiding reinfection

Multiple interviewees attributed their HCV acquisition primarily to associating with peers in high‐risk environments rather than their personal engagement in risk behaviours. Gay community venues and the sexual and social networks of interviewees were portrayed as risk‐perpetuating environments, where higher risk sexual practices were common and drug use was perceived as ubiquitous:

*My idea of the gay community got replaced by this, the whole public that use drugs. Which is probably half the reason I'm fearful for kind of getting back into that because I'm still not sure how to be sober within that* (Liam, 26).

Interviewees commonly described being introduced to methamphetamine or invited to inject the drug by a sexual partner. Some participants described seeking out people with similar drug habits, in part to avoid judgement and negative self‐appraisal resulting from the social unacceptability of methamphetamine use, and spoke about selecting sexual partners to facilitate drug use: *It kind of becomes this thing where you no longer know whether you're looking for sex for the sex or whether you're looking for sex for the drug*. (Joshua, 38)

Interviewees who used methamphetamines spoke about being rejected by friends who disapproved of these practices. Lifestyle changes catalysed by HCV treatment therefore resulted in periods of loneliness and isolation as interviewees disengaged from drug‐using social networks: *I was pretty lonely…But I had to stay in (at home) and I knew it* (Dan, 34). Those who maintained relationships within drug‐using networks were mindful of the challenges these environments presented:

*I've been clean since* [the beginning of HCV treatment]. *So it's been pretty good but hard, only because now I gotta change my circle of friends again because… it's difficult, they'll be still using when I'm with them* (Oliver, 44).

### 3.4 HCV care as a catalyst for change

Participation in the co‐EC trial was described as an opportunity to cure a life‐threatening illness and reducing anticipated stigma, accompanied by the relief of having *one less thing to deal with* (Marcus, 46). While the availability of effective HCV treatment made some participants *less concerned about the implications (of reinfection)* (Angelo, 47), everyone expressed commitment to staying HCV‐free. This was partially motivated by the fear of being labelled an injecting drug user and having to disclose HCV:

*In a way I'm glad the stigma does stick with me in the sense that I don't want to have the stigma of having it. I don't want to have the stigma if that* [my partner] *and I were no longer going out with each other it would mean I'd have to get back on that merry‐go‐round (of disclosure). And I don't want to get reinfected*. (Geoff, 48)

Engagement in HCV care contributed to improved competencies around HCV transmission risk reduction. Interviewees said they intended to ask prospective sexual partners about their HCV serostatus and avoid condomless, mucosally abrasive sex with partners whose HCV serostatus was unknown and who were known to be injecting drugs. It was observed that *people that have had hep C and finished the treatment…are more open about it once they don't have it anymore* (Dan, 32), but discussing HCV with sexual partners was described as challenging:

*Now I've been asking people* [about hep C] *and some people get a bit offended*. … *I go, “I wasn't making it sound like you were a user, it was just in case.” We're still gonna use a condom anyway, it's just one of those precautions* (Oliver, 44).

Beyond motivating an enhanced awareness and practice of harm minimization, several interviewees described their engagement in HCV care as a catalyst for more significant changes that resulted in reducing their use of methamphetamines and engagement in sexualized drug use:

[The treatment] *set me up with supports that I could use to change my life…if I wanted to change my life, now would be a good time to do it because I'm gonna have a lot of knowledgeable people in it who will support me through things* (Dylan, 47).

*That's why I wanted to stop using* [drugs]. *I'm too scared, in case I get reinfected. Because to me that would be a waste of time* (Oliver, 44).

For some, HCV reinfection was framed in terms of a failure to maintain abstinence. They perceived risk as rooted in drug‐using social environments and prevention strategies therefore centred on avoiding certain social networks to circumvent drug exposure and temptation to use:

*Because I've stopped using besides the occasional relapse it's clear that I don't want ice (methamphetamine) in my life. So that makes it much easier* [to avoid reinfection], *because I'll be much less in any circle, close to circles at higher risk* (Joshua, 38).

Interviewees who continued to use methamphetamines expressed confidence about practising better drug‐related risk reduction. Some regarded themselves as advocates for peer education within drug‐using networks: *I like to communicate all this stuff with people that also do the same drug. That way we make things safer for ourselves*. (Michael, 48)

## 4 Discussion

We explored the experiences, attitudes and beliefs of GBM living with diagnosed HIV who had recently been treated and cured of HCV. Participants typically described a lack of acknowledgement of HCV and associated risk which was fuelled by an “othering” of HCV as a disease belonging to heroin injectors and related specifically to injecting risk. Their lack of awareness of sexual transmission of HCV, which is how most believed they acquired the virus, resulted in participants blaming their infection on a combination of lack of awareness, high‐risk environments and sexual networks, rather than their personal engagement in risk behaviours. The explicit connection made by participants between HCV exposure in sexual environments and ‘sexualized’ drug use resulted in participants framing their desire to avoid reinfection in the context of maintaining abstinence from drugs and disengaging from drug‐using social networks. In this way, GBM shared commonalities with PWID in terms of acknowledging the need to modify drug‐using behaviours to avoid HCV reinfection. However, the desire of GBM to avoid drug use – beyond some participants being at a stage of maturing out of drug use – was related to its association with sexual risk as opposed to injecting‐related risk. These insights suggest that strategies aimed at reducing post‐treatment HCV reinfections among GBM must address a range of factors that characterize HCV risk environments for GBM, including the use of drugs as both direct and indirect drivers of transmission.

The lack of HCV knowledge and awareness – in particular among drug‐injecting GBM – may seem surprising, as GBM in Australia are generally described as risk aware in the context of HIV 47, 48. This discordance in risk awareness may reflect the stigma associated with HCV infection and injecting drug use, with respondents trying to differentiate themselves and their practices from “more entrenched” PWID and heroin‐injecting drug users in particular. This distinction between types of drug use and types of drug users is consistent with findings from research conducted among gay methamphetamine‐using men in Sydney 49, which elucidated the differing norms and levels of social acceptability of certain drugs and drug practices. A hierarchical organization of multiple stigma has also been described among PLHIV 50, 51 and highlighted in the context of HCV/HIV co‐infection 52, confirming that HCV infection can be experienced as more stigmatizing than HIV among GBM. This has clear implications for HCV risk through the non‐disclosure of HCV infection, compromising the perceived “safety” of HIV‐serosorted and condomless sexual practices commonly found within chemsex communities 53, 54, 55. With the relatively recent emergence of HCV as a sexually transmitted infection in the context of HIV, it is unclear whether discrimination by HIV healthcare professionals played a role in stigma experienced by co‐infected GBM. However, clinicians practising in the specialist clinics from where our sample was recruited were generally experienced in providing HCV care and others have noted that clinicians with increased contact with patients living with HCV often hold more favourable attitudes towards PWID 56. Depending on local context, health professionals can be both a source of stigma 51 and a vehicle for stigma reduction 57.

Interviewees reported seeking out people similar to them in terms of HIV serostatus and drug use which may reflect a form of psychological risk management to avoid anticipated stigma and negative self‐perception 58, 59, given participants also spoke about rejection by other friends because of their drug‐using behaviours. Close engagement with peers living with diagnosed HIV and people who use drugs led to more time spent in risk environments. To avoid reinfection participants often chose to disengage from risk environments and attain abstinence, which meant isolation from social networks and loneliness for some interviewees. With fear of isolation potentially resulting in some GBM remaining in or returning to risky sexual networks, equipping GBM with accurate knowledge about HCV transmission risks as a part of clinical care provides an important HCV prevention opportunity. Acknowledging that improving HCV knowledge only may not produce the levels of behaviour change required to achieve elimination in the co‐EC study or more broadly 60, 61, effective risk reduction interventions are also likely to require the construction of supportive community environments, including through peer group social norms 62. Healthcare‐engaged members of at‐risk communities can act as partners in intervention delivery through endorsement and enactment of prevention practices 63 and through the peer distribution of risk reduction equipment (including gloves, lubricant, sterile needles and sharps containers) at private sex parties or sex‐on‐premises venues. As reflected in the discussions with our interviewees, engagement in HCV care had contributed to improved competencies and an intention to more openly discuss HCV. Diffusion of HCV risk awareness through GBM treated and cured could help to destigmatize HCV and empower GBM to address HCV among their sexual and drug‐using networks.

When discussing HCV risk reduction practices with GBM living with diagnosed HIV, healthcare providers should consider several social factors. The potential conflict between a wish to maintain a lifestyle that reduces health risks and fear of social isolation as a result of risk management may present a considerable challenge to reinfection avoidance. While the literature reports good community support for GBM living with diagnosed HIV 64, support for people with HCV infection is often primarily tailored towards opioid‐using PWID 65, 66, 67. Establishing supportive peer networks for co‐infected GBM and those who have recently cleared their HCV infection could mitigate the loss of social capital following disengagement from previous networks and enable positive reinforcement through shared experiences.

## 5 Limitations

There are some limitations to this study. The potential for social acceptability bias must be acknowledged, with some participants possibly describing their commitment to HCV reinfection avoidance more positively. Similarly, interviewer bias may have led participants to be less open about their experiences in front of a young, heterosexual woman; however, being interviewed by an outsider may have allowed others to speak more freely. Furthermore, our sample was comprised only of GBM engaged in HIV care who had already undergone HCV treatment, possibly excluding members of the population at higher risk of ongoing HCV transmission whose attitudes might differ. Finally, the limited time available to conduct this study did not allow the theoretical sampling period typically included in grounded theory studies 45. Nevertheless, use of constructivist grounded theory as a research approach has facilitated an in‐depth understanding of the social processes underlying HCV risk among GBM.

## 6 Conclusions

This study illustrates that HCV infection among GBM is experienced as both a behavioural and social outcome. Our findings suggest that adoption and reduction in risk behaviours emerges from the complex interplay between individuals and the (social) environments they find themselves in, as well as personal motivations and individual ability to identify and act upon specific risks. HCV prevention campaigns tailored towards GBM living with diagnosed HIV need to take account of the multiplicity of transmission risks in the context of sexualized drug use and the intersectionality of multiple stigmatized social identities. Engagement in HCV care presents an important opportunity to provide support beyond curing HCV and could be pivotal in facilitating the behaviour change necessary to achieve elimination targets. GBM choosing to disengage from their communities as a form of risk management may be stifled by a loss of social capital which could present a long‐term barrier to avoiding HCV reinfection. Healthcare practitioners providing treatment support should be mindful of this potential conflict between a desire to remain HCV uninfected and in control of drug use and a wish for belonging and social integration. Establishing supportive peer networks among GBM with a shared experience of HCV infection could alleviate social capital loss and mitigate HCV reinfection risks.

## Competing interests

The co‐EC study was funded by an investigator initiated research grant from Bristol Myers Squibb. JD and MH's institution receives investigator initiated research funding from Abbvie, Merck, Gilead Sciences and Bristol Myers Squibb. JD and his institution have received consultancy fee/honoraria from AbbVie, Merck, Gilead Sciences and Bristol Myers Squibb. PH receives investigator initiated research funding from Gilead Sciences and Abbvie for research not related to this study. The authors declare no other potential conflicts of interest.

## Authors’ contributions

All authors have read and approved the final manuscript. S.S., P.H., M.S. and J.D. performed the research. M.S., J.D., M.H. and S.S. designed the research study. P.H., G.B. and A.P. contributed to the development of the interview schedule. R.W. coordinated and facilitated the recruitment. S.S., M.S. and P.H. analysed the data. S.S. and M.S. were primary writers of the paper.
